# Low Soil Nutrient Tolerance and Mineral Fertilizer Response in White Guinea Yam (*Dioscorea rotundata*) Genotypes

**DOI:** 10.3389/fpls.2021.629762

**Published:** 2021-02-19

**Authors:** Ryo Matsumoto, Haruki Ishikawa, Asrat Asfaw, Robert Asiedu

**Affiliations:** International Institute of Tropical Agriculture, Ibadan, Nigeria

**Keywords:** *Dioscorea*, low soil fertility tolerance, soil fertility susceptibility, West Africa, genotypic variation

## Abstract

Yam (*Dioscorea* spp.) is a major food security crop for millions of resource-poor farmers, particularly in West Africa. Soil mineral deficiency is the main challenge in yam production, especially with the dwindling of fallow lands for the indigenous nutrient supply. Cultivars tolerant to available low soil nutrients and responsive to added nutrient supply are viable components of an integrated soil fertility management strategy for sustainable and productive yam farming systems in West Africa. This study’s objective was to identify white Guinea yam (*D. rotundata*) genotypes adapted to available low soil nutrients and responsive to externally added nutrient supply. Twenty advanced breeding lines and a local variety (Amula) were evaluated under contrasting soil fertility, low to expose the crop to available low soil nutrient supply and high to assess the crop response to added mineral fertilizer (NPK) input at Ibadan, Nigeria. The genotypes expressed differential yield response to low soil fertility (LF) stress and added fertilizer input. Soil fertility susceptibility index (SFSI) ranged from 0.64 to 1.34 for tuber yield and 0.60 to 1.30 for shoot dry weight. The genotypes R034, R041, R050, R052, R060, R100, and R125 combined lower SFSI with a low rate of reduction in tuber yield were identified as tolerant to LF stress related to the soil mineral deficiency. Likewise, the genotypes R109, R119, and R131 showed high susceptibility to soil fertility level and/or fertilizer response. Genotypes R025 and R034 had the tuber yielding potential twice of that the local variety under low soil nutrient conditions. Shoot dry weight and tuber yield showed a positive correlation both under low and high soil fertility conditions (*r* = 0.69 and 0.75, respectively), indicating the vigor biomass may be a morphological marker for selecting genotypes of white Guinea yam for higher tuber yield. Our results highlight genotypic variation in the tolerance to low soil nutrients and mineral fertilizer response in white Guinea yam to exploit through breeding and genetic studies to develop improved genotypes for low and high input production systems in West Africa.

## Introduction

Yam (*Dioscorea* spp.) is an important tuber crop for human consumption. Yam is a common name of the various species in the genus *Dioscorea* with varying origins and distributions in the tropics, subtropics, and temperate regions (Darkwa et al., 2020). It is a highly valuable crop in the food and cultural systems of West Africa (Asiedu and Sartie, 2010), where 66.8 million tons (93%) of the global production occurs (FAOSTAT, 2020). Among the cultivated species, the white Guinea yam (*D. rotundata*) is the most planted, produced and consumed yam in West Africa (Asfaw et al., 2020). The yam production in West Africa has increased from 14.5 million tons in 1988 to 66.8 million tons in 2018. The annual rate of increase in total yam production was around 3.8% from 2000 to 2019 (FAOSTAT, 2020). The dramatic increases are associated with area expansion into the Savannas. However, chemical inputs are limited, and landraces are still frequently used in yam cultivation in West Africa (Degras, 1993; Scott et al., 2000). The fallow period traditionally lasted 20 years in the forest-savannah agro-ecological zone of West Africa. Recently, the fallow period’s duration has decreased to less than five years because of population pressure and land competition from other crops and purposes (Ouédraogo, 2004; Asiedu and Sartie, 2010). A sharp decline in yam yield occurs when grown after a limited fallow of approximately 1–3 years (Watson and Goldsworthy, 1964). The yam yield has declined from 9.4 t ha^–1^ in 1988 to 8.4 t ha^–1^ in 2018 (FAOSTAT, 2020). Although there was productivity growth, it has been relatively marginal when compared to potato (FAOSTAT, 2020). This trend could be catastrophic unless steps are taken to change the situation (Manyong and Nokoe, 2003).

Several soil nutrient management technologies that enhance crop productivity have been developed, tested, and promoted in Africa’s low-input farming systems (Kihara et al., 2020). However, there is little information on yam’s input intensification technologies (Enesi et al., 2017). The results and recommendations of the few available soil fertility research on yams are variable and sometimes conflicting (Irving, 1956; Lugo et al., 1993; O’Sullivan and Ernest, 2008; Diby et al., 2009; Carsky et al., 2010; Enesi et al., 2017). Irving (1956) reported a positive tuber yield response of yam to N fertilizer application in eastern Nigeria. Likewise, Kpeglo et al. (1981) in southwest Nigeria and Lyonga (1981) in the northwest highlands of Cameron reported tuber yield increase with mineral fertilizer. In contrast, Carsky et al. (2010) reported no yield response to N mineral fertilizer application in Togo. However, their study showed tuber yield increases with K and tuber size increase with P application. Kang and Wilson (1981) also reported no significant chemical fertilizer effect on yam tuber yield. Diby et al. (2009) and Lugo et al. (1993) indicated a differential yield response to chemical fertilizer application in yam, depending on soil fertility conditions. The studies on the mineral fertilizer effect on yam were either used few cultivars or without creating nutrient-depleted plots to correctly assess the impact. However, fertilizer impact studies clearly highlighted promoted growth and yield of yam under low fertile soils (Diby et al., 2011).

Intensification of yam production through soil amendments, improved cultivars, and other inputs could bring yield increases per unit area. Cultivars tolerant to available low soil nutrients and responsive to external nutrient supply are the most effective and convenient way to improve productivity in low input small-holder farming systems. Cultivar tolerance to low soil nutrients supports sustainable crop production by reducing production costs and farmer dependence on fertilizers. On the other hand, cultivar responsive to mineral fertilizer application aids crop intensification to enhance production. However, studies on genetic variation to low and high soil fertility in yam crops in general and white Guinea yam are scarce. Genetic improvement efforts need to integrate high yields with high nutrient use efficiency (Asiedu and Sartie, 2010). Information on genetic variation within yam germplasm to low and high nutrient availability is a stepping stone to a selective breeding to improve yam for low and high input farming systems. Therefore, this study assessed genotypic differences among white Guinea yam (*D. rotundata*) genotypes for improved productivity under low and high soil nutrient conditions.

## Materials and Methods

### Site and Soil Properties

The field experiments were conducted for two years (2018 and 2019 cropping seasons) at the low soil fertility (LF) experimental field at the International Institute of Tropical Agriculture (IITA), 7°31′ N and 3°54′ E, southwestern Nigeria. The LF condition was created by continuously cultivating high nutrient mining crops such as cassava, maize, and sorghum without fertilizer input from 2016 to 2018. Trial plots with the dimensions 100 m × 25 m were used. Cassava was grown from April 2016 to March 2017, followed by sorghum from April 2017 to January 2018, and then maize for four consecutive harvests from April 2017 to January 2018. The continuous planting of high nutrient mining crops in the same plot without additional nutrients successfully created LF or mineral nutrient-deficient soil conditions for our experiment.

Soil samples were collected in April 2016 and March 2018 at depths of 0 – 20 cm from thirty randomly selected spots in the plot. Samples were dried at 65°C before sifting (using a 2 mm sieve). Soil pH was determined by initially suspending the soil in water (at a 1:2.5 soil/water ratio). Exchangeable Ca^2+^, Mg^2+^, K^+,^ and available P were extracted according to the Mehlich-3 procedure (Mehlich, 1984). The cations were determined using an atomic absorption spectrophotometer (Accusys 211 Atomic Spectrophotometer, Buck Scientific, CT, United States). Phosphorus was assayed colorimetrically using Genesys 10S UV-Vis (Thero Scientific, MA, United States). Organic carbon was determined by chromic acid digestion with a spectrophotometric procedure using the Genesys 10S UV-Vis (Heanes, 1984). Total nitrogen was determined using the Kjeldahl method for digestion and colorimetric determination on a Technicon AAII Autoanalyzer (Seal Analytical, WI, United States) (Bremner and Mulvaney, 1982). The soil properties are presented in Table 1. In addition, the weather data for the experimental period was assessed using the data obtained from the Geographical Information System (GIS) unit of IITA.

**TABLE 1:** Soil chemical properties of the trial sites at IITA Ibadan, Nigeria.

**Soil Properties**	**2016**	**2018**
pH	5.93	6.41
Organic carbon (%)	1.23	0.15
Nitrogen (%)	0.13	0.03
Phosphorus (mg kg^–1^)	6.12	1.62
Calcium (Cmol[+] kg^–1^)	3.36	3.19
Magnesium (Cmol[+] kg^–1^)	0.46	0.90
Potassium (Cmol[+] kg^–1^)	0.59	0.27

### Plant Materials and Trial Management

Twenty advanced genotypes from the IITA yam breeding program and a popular landrace cultivar (Amula) constituted the plant material for this study (Supplementary Material 1). All genotypes used for the field experiment were multiplied under uniform conditions at the IITA field in the 2017 cropping season to generate quality planting material. The plants with symptoms of virus disease such as yam mosaic virus were removed from field during the growing period. Visually assessed clean tubers with no sign of rot and pests were used as seed tuber material for planting the trials. Tubers weighing approximately 1 - 2 kg were cut horizontally to remove the head and tail components. The tuber’s center component was cut into pieces of 50 ± 10 g to obtain uniform material for planting (yam setts). Yam setts were treated in a mixture of 70 g mancozeb (fungicide) and 75 mL chlorpyrifos (insecticide) in a 10 L volume of tap water for 5 min, and then the setts were dried for 20 h in the shade before planting for pre-sprouting. The yam setts were planted in plastic pots (12 cm diameter × 10 cm height) filled with topsoil. Plants with uniform sprouts were transplanted into the field with stakes 30 days after planting. In 2018 and 2019 trials, trial plots with dimensions 25 m × 25 m were prepared in LF soil plots for screening, respectively. The field experiment consisted of a split-plot in randomized block design replicated twice (Altman and Krzywinski, 2015). The main plot constituted two levels of fertilizer applications, nil as Low soil fertility condition and 90, 50, 75 kg N, P, and K ha^–1^ as high fertility soil condition (HF), while the subplot consisted of 21 genotypes. All plants in the main plot were at the planting density of 1 plant m^–2^. Each subplot had 3 plants with a plot size of 3 × 1 m^2^ without a border plant. The main plot size was 3 × 21 m^2^ and replicated twice. There was three meters channel between replications. The ten meters channel was created between non-fertilized and fertilized plot (detail field design presented in Supplementary Material 2). Fertilizer was applied at 14 days after transplanting using the side dressing method. Weeds were removed manually whenever present to maintain weed-free plots throughout the experiment.

### Data Collection

Shoot dry weight was evaluated using the non-destructive method described in Iseki and Matsumoto (2019) at 150 days after planting (150 DAP). This assessment correlated with yam’s maximum aerial growth (Diby et al., 2011; Sartie et al., 2012). The normalized difference vegetation index (NDVI) was measured using a handheld sensor (GreenSeeker, Nikon Trimble, Tokyo, Japan) with a simultaneous plant height and green area measurement. Shoot dry weight was estimated by an equation using NDVI and plant height as explanatory variables. Shoot dry weight was evaluated for the three plants in each plot. In December, when the aerial parts were at full senescence, each plant’s tubers were harvested at 230 DAP in the first-year and 232 DAP in the second-year. After harvesting, the fresh tuber weight was recorded. Biomass reduction in shoot dry weight at 150 DAP and tuber yield was calculated as

Shootbiomassandtuberyieldreductionrate(%)

$$\;=\left(\frac{\bar{\mathrm{xhf}}-\bar{\mathrm{xlf}}}{\bar{\mathrm{xhf}}}\right)\times 100$$

where $\bar{xlf}$ and $\;\bar{xhf}$ are the mean trait values of a given genotype under low and high soil nutrient environments, respectively.

The soil fertility susceptibility index (SFSI) was calculated using the following formula (Fischer and Maurer, 1978) and defined as

$$S\,F\,S\,I=\left(1-\frac{\bar{x\,l\,f}}{\bar{x\,h\,f}}\right)/\left(1-\frac{\bar{Y\,l\,f}}{\bar{Y\,h\,f}}\right)$$

where $\bar{xlf}$ and $\;\bar{xhf}$ are the mean trait values of a given genotype under low and high nutrients soil environments, respectively. $\bar{Ylf}$ and $\bar{xhf}$ are the mean trait values of all genotypes under low and high soil nutrients environments, and $\;1-\bar{Ylf}/\bar{Yhf}$ is the soil fertility intensity (SI).

### Data Analysis

Data were analyzed using the linear mixed model in lme4 package (Bates, 2010) in the R environment for statistical computing (R Core Team, 2018). To determine if there is a significant difference between the means value of trait obtained from LF and HF condition for each genotype, T-test was performed using R package ggplot2 (Kassambara, 2020). To compare the shoot growth and tuber yield performance of improved genotypes with local variety under LF condition, Dunnett’s test was performed using multcomp R package (Hothorn et al., 2020). Person’s correlation coefficients among traits were calculated using R package *corregram* (Wright, 2018).

## Results

### Status of Soil Fertility and Meteorological Condition in the Experimental Field

The soil chemical property of the experimental field is presented in Table 1. Planting cassava, sorghum, and maize before yam planting depleted mineral nutrients and created low soil fertility stress for the yam experiment. The soil pH was 5.93 in 2016 and 6.41 in 2018. The organic carbon content was 1.23% in 2016. However, the organic carbon content was decreased to 0.15% when the field trial was set up for yam in 2018. The nitrogen content in 2018 (0.03%) was lower than in 2016 (0.13%). The available phosphorus content was 6.12 mg kg^–1^ in 2016, and it was reduced to 1.62 in 2018. The exchangeable Ca^2+^ and Mg^2+^ in 2016 were 3.19 Cmol[+] kg^–1^ and 0.46, Cmol[+] kg^–1^, respectively. In 2018, the exchangeable Ca^2+^ and Mg^2+^ were 3.19 Cmol[+] kg^–1^ and 0.90 Cmol[+] kg^–1^, respectively. The exchangeable Ca^2+^ in 2016 and 2018 was 3.19 and 3.97 Cmol[+] kg^–1^, receptivity. The exchangeable Mg^2+^ was 0.46 Cmol[+] kg^–1^ in 2016 and 0.90 Cmol[+] kg^–1^ in 2018. Exchangeable K^+^ was 0.59 Cmol[+] kg^–1^ in 2016, which was higher than in 2018 (0.27 Cmol[+] kg^–1^).

**FIGURE 1:**
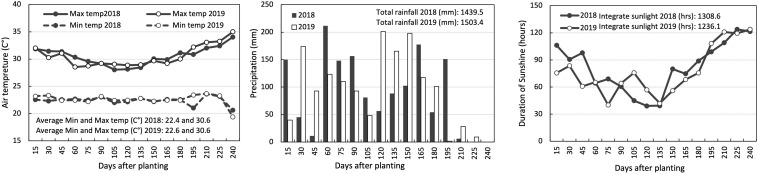
Air temperature, precipitation, and duration of sunshine during the growth periods at IITA Ibadan, Nigeria. The air temperature (left box) includes solid lines and dashed lines representing maximum and minimum air temperature averages, respectively, for every 15 days after planting. For precipitation (center box) and sunshine duration (right box), data are cumulative values for every 15 days after planting.

Figure 1 presents the meteorological conditions during the growth period, from planting to harvest (240 days after planting) for the trials. The total precipitation, average minimum/maximum temperatures, and integrated sunshine duration for this period were 1439.5 mm, 22.4/30.6°C, 1308.6 h in 2018, and 1503.4 mm, 22.6/30.6°C, and 1236.1 h in 2019.

### Effect of Soil Fertility on Shoot Dry Weight and Tuber Yield

**FIGURE 2:**
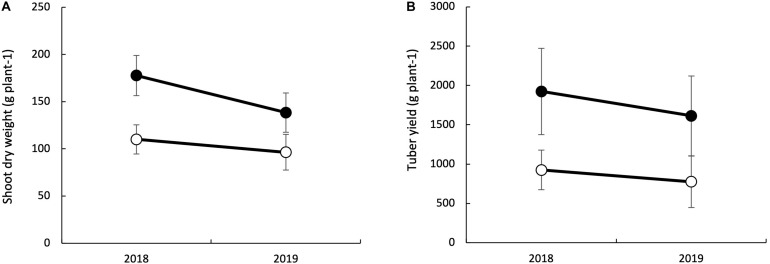
Effects of fertilizer application on shoot dry weight at 150 days after planting **(A)** and tuber yield **(B)** of white Guinea Yam varieties. Bars indicate the standard deviation (*n* = 21).

The main effects of genotype, soil fertility treatment, and year were significant for tuber yield and shoot dry weight (Table 2). The interaction effects were significant between a year and soil fertility treatments on shoot dry weight. The gap in shoot dry weight between LF and HF conditions was enormous in 2018 than in 2019. On the other hand, the interaction of genotype with soil fertility status was significant for tuber yield. Mean shoot dry weight and tuber yield were higher in 2018 than in 2019 (Figure 2). The overall mean shoots dry weight increased by 34.7% with HF condition, while such an increase was 51.9% for tuber yield.

**TABLE 2:** Analysis of deviance (Type II Wald chi square tests for linear mixed model) of shoot dry weight and tuber yield in 21 white Guinea yam genotypes.

**Shoot dry weight**	**Chisq**	**Df**	**Pr (> Chisq)**	
Year (Y)	30.6	1	1.1E−12	***
Fertilizer treatment (T)	261.1	1	2.2E−16	***
Genotype (G)	63.5	20	8.0E−01	***
Y x T	20.9	1	54E−06	***
Y x G	17.7	20	1.00	
T x G	28.6	20	0.14	
Y x T x G	24.9	20	0.72	
Tuber yield				
Year (Y)	11.6	1	0.001	***
Fertilizer treatment (T)	183.9	1	2.2E−16	***
Genotype (G)	147.9	20	2.2E−16	***
Y x T	1.4	1	0.24	
Y x G	28.7	20	0.09	
T x G	67.3	20	0.5E−06	***
Y x T X G	16.8	20	0.67	

*** Represents significant differences at *p* < 0.001.

**FIGURE 3:**
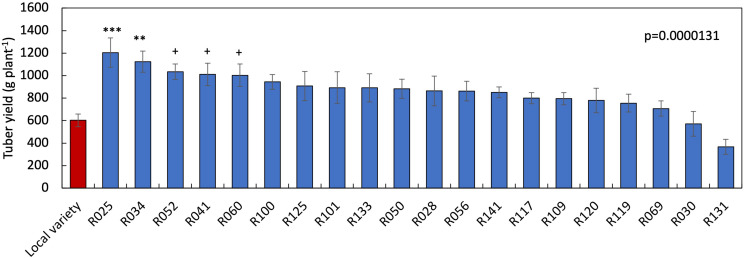
Varietal differences in tuber yield in 21 white Guinea yam genotypes at low nutrients soil conditions. Data are the average of values obtained during a 2-year field experiment. Statistical significance was determined by Dunnett’s test. ***(*p* < 0.001), **(*p* < 0.01) and + (*p* < 0.1) when compared with the local variety. Bars indicate standard error (*n* = 12). R means TDr1302 as IITA yam breeding code.

Among the genotypes screened under two-variable soil fertility status, sixteen produced significantly higher tuber yield in HF condition than when grown with LF condition (Table 3). In contrast, five genotypes did not show significant tuber yield differences between the LF and HF conditions. The percent tuber yield reduction due to LF stress ranged from 33.4 (R125) to 69.8% (R119). The SFSI in tuber yield ranged from 0.64 to 1.34, with a mean of 0.98. The genotypes R034, R041, R050, R052, R060, R100 and R125 showed a lower SFSI than the local variety (0.87) (Table 3). On the other hand, R109, R119, and R131 showed a higher SFSI and larger reduction rate among tested genotypes. Tuber yield ranged from 367.5 (R131) to 1205.0 (R025) g plant^–1^ under LF condition (Figure 3). The tuber yield of R025 and R034 were significantly higher than that of the local variety (199% and 187%, respectively) followed by R052 at LF condition.

**TABLE 3:** Effect of genotype and soil fertility on tuber yield (gram per plant) in 21 white Guinea yam genotypes.

**Genotype**	**Tuber yield (g plant^–1^)^†^**				
	**LF**	**HF**	**PR**	***p*-Value**	**SFSI**
R125^†⁣†^	908.3	1363.8	33.4	0.05	0.64
R052	1034.6	1604.2	35.5	0.10	0.68
R060	1003.3	1587.5	36.8	0.09	0.71
R041	1010.8	1652.5	38.8	0.08	0.75
R100	944.2	1639.2	42.4	0.18	0.82
R050	882.9	1545.0	42.9	0.04	0.83
R034	1124.2	2028.3	44.6	0.01	0.86
Local variety	602.9	1099.2	45.1	0.01	0.87
R133	891.7	1775.8	49.8	0.04	0.96
R069	707.5	1419.4	50.2	0.01	0.97
R120	780.0	1572.5	50.4	0.00	0.97
R101	893.3	1860.0	52.0	0.01	1.00
R141	851.7	1842.5	53.8	0.01	1.04
R117	800.0	1799.2	55.5	0.03	1.07
R028	863.8	1974.2	56.2	0.00	1.08
R025	1205.0	2756.7	56.3	0.00	1.08
R056	863.3	1992.5	56.7	0.01	1.09
R030	571.7	1461.7	60.9	0.00	1.17
R109	795.0	2506.7	68.3	0.00	1.32
R131	367.5	1160.0	68.3	0.01	1.32
R119	755.8	2500.0	69.8	0.01	1.34
Mean	850.4	1768.6	50.8		0.98

*^†^LF, low soil fertility; HF, high soil fertility; PR, present reduction due to LF; SFSI, Soil fertility susceptibility index; *p*-value was calculated using *t-test* between low soil fertility and high soil fertility condition. R means TDr1302 as IITA yam breeding code. Detail codes for the genotypes presented in Supplementary Material 1.*

The shoot dry weight of fourteen genotypes was significantly lower in LF than HF conditions (Table 4). On the contrary, seven genotypes showed non-significant differences in shoot dry weight between the LF and HF conditions (Table 4). Genotype R131 showed a 44.9% reduction in shoot dry weight in LF condition than the HF condition (averaged across the years). The lowest shoot dry weight reduction of 29.2% was recorded from the genotype R125 (Table 4). The soil fertility susceptibility index (SFSI) for shoot dry weight ranged from 0.64 (R125) to 1.30 (R131). The genotypes R041, R052, R101, R125, and R141 had lower SFSI values than the local variety (0.88).

**TABLE 4:** Effect of genotype and soil fertility on shoot dry weight (gram per plant) in 21 white Guinea yam genotypes.

**Genotype**	**Shoot dry weight (g plant^–1^)^†^**				
	**LF**	**HF**	**PR**	***p-*Value**	**SFSI**
R125	110.3	142.5	22.6	0.06	0.64
R101	107.4	139.0	22.7	0.16	0.66
R052	108.3	143.9	24.8	0.14	0.71
R041	127.0	172.6	26.4	0.06	0.76
R141	113.3	153.9	26.3	0.05	0.76
Local variety	104.1	150.4	30.8	0.06	0.08
R060	97.7	142.5	31.4	0.10	0.89
R120	101.9	152.2	33.1	0.03	0.95
R100	104.4	159.4	34.5	0.04	0.97
R028	113.1	171.7	34.1	0.00	0.99
R069	92.9	142.3	34.7	0.02	1.00
R025	116.4	177.9	34.6	0.05	1.00
R133	102.3	164.2	37.7	0.01	1.08
R117	100.9	162.3	37.8	0.03	1.09
R034	101.8	167.4	39.2	0.01	1.12
R050	98.6	163.6	39.7	0.02	1.14
R056	98.0	164.5	40.4	0.01	1.16
R119	99.3	170.6	41.8	0.04	1.20
R030	84.9	148.7	42.9	0.01	1.24
R109	106.3	188.1	43.5	0.00	1.25
R131	80.1	145.4	44.9	0.02	1.30
Mean	103.3	158.2	34.7		0.99

*^†^LF, low soil fertility; HF, high soil fertility; PR, present reduction due to LF; SFSI, Soil fertility susceptibility index. *p-*Value was calculated using *t-test* between low soil fertility and high soil fertility condition. R means TDr1302 as IITA yam breeding code. Detail codes for the genotypes presented in Supplementary Material 1.*

**FIGURE 4:**
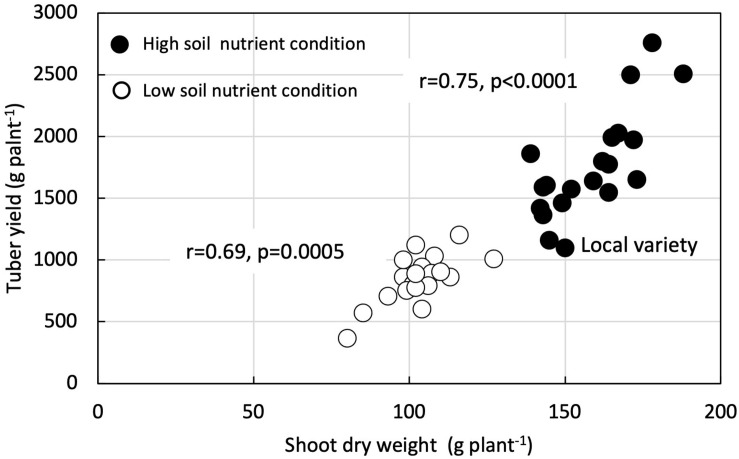
Correlation between shoot dry weight and tuber yield under low nutrients soil fertility condition and high nutrient soil condition in 21 white Guinea yam genotypes.

**FIGURE 5:**
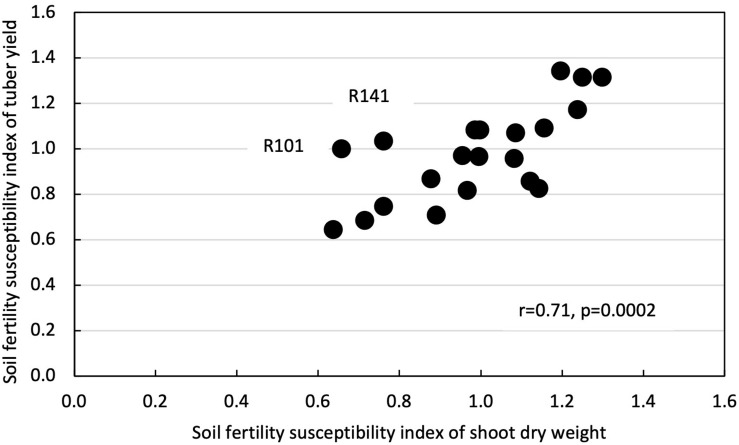
Correlation between soil fertility susceptibility index of shoot dry weight and soil fertility susceptibility index of tuber yield in 21 white Guinea yam genotypes. R means TDr1302 as IITA yam breeding code.

Shoot dry weight had a positive and significant correlation with tuber yield in both LF (*r* = 0.69, *p* = 0.0005) and HF plots (*r* = 0.75, *p* < 0.0001) (Figure 4). Likewise, SFSI for shoot dry weight had a positive and significant correlation with SFSI for tuber yield (*r* = 0.71, *p* = 0.0002) (Figure 5).

## Discussion

This study assessed white Guinea yam genotypes’ response to contrasting soil fertility levels, the LF and HF at field condition. The LF condition created by planting cassava, sorghum, and maize before yam planting was significant for assessing the yam’s genetic variation at variable soil nutrients. Due to cassava, sorghum and maize cultivation from 2016 to 2018, the nitrogen, phosphorus and potassium contents decreased by 0.10%, 4.50% and 0.32 Cmol[+]kg^–1^, respectively (Table 1). Cassava is heavy soil nutrient mining crop that could remove up to 86 - 177 kg ha^–1^ nitrogen, 70 - 104 kg ha^–1^ phosphorus and 70 - 104 kg ha^–1^ potassium from the soil during the growing period (Ezui et al., 2017). Likewise, Kumar et al. (2017) reported that sorghum plant could remove up to 107 - 290 kg ha^–1^ nitrogen, 62 -152 kg ha^–1^ phosphorus and 339 - 731 kg ha^–1^ potassium from the soil during growing period. Heckman et al. (2003) has quantified the nutrient removal by maize grain as 10.2 to 15.0 g kg^–1^ of nitrogen, 2.2 to 5.4 g kg^–1^ of phosphorus and 3.1 to 6.2 g kg^–1^ potassium. Soil mineral nutrient depletion by planting cassava, sorghum and maize in our experimental field was substantial. According to Chude et al. (2011), our experimental field (Table 1) had available low soil nutrients and could be inferred as close to a critical level for yam cultivation. Though the critical soil nutrient levels for yam cultivation has not yet established in West Africa, yam cultivation in soils containing < 0.1% nitrogen, < 10 mg kg^–1^ available P, and 0.15 Cmol[+] kg^–1^ of exchangeable K requires external fertilizer inputs (Carsky et al., 2010).

Yam tubers express variable sprouting time depending on the position or section of the tuber cut as a sett and used for planting. Seed tuber setts cut from the head section often sprouts earlier than that cut from the middle and tuber tail parts (Onwueme, 1973). The variation in sprout emergence time is the main cause of variation in shoot biomass size and tuber yield within plot in yam trials (Cornet et al., 2014). Within plot tuber yield variation due to differential sprout emergence of the tuber setts originating from the different section of the tuber is a potential factor complicating the interpretation of the field experiment results in yam. To control such variability, this study used setts from the middle section tubers as planting material and pre-sprouted them to successfully establish uniform plants within plots in the field and assess genotypes response to low soil fertility stress and added fertilizer input.

We identified genotypes sensitive and tolerant to low nutrient soil stress using the susceptibility index values (Tables 3, 4). The susceptibility index has been widely used to identify sensitive and tolerant genotypes for drought stress in wheat (Fischer and Maurer, 1978; Clarke et al., 1984; Winter et al., 1988; Clarke et al., 1992), maize, triticale (Kutlu and Kinaci, 2010; Grzesiak et al., 2013), and cassava (Oliveira et al., 2017) and soil fertility stress in common bean (Singh et al., 2003). Accordingly, R119, R109, and R131 combined higher SFSI values with tuber yield increase at HF condition were suitable candidates to maximize productivity under high input cultivation. Likewise, R034, R041, R050, R052, R060, R100, and R125 combined lower SFSI with a low rate of reduction in tuber yield were tolerant to low soil fertility stress. The genotype R025 and R034 had the tuber yielding potential twice of local variety under low nutrient soil stress (Figure 3). Besides, R025 has the yield potential of more than 1 kg of tuber plant^–1^ under the LF condition, which is higher than that has been reported in previous white Guinea yam study (Sartie et al., 2012).

There have been many conflicting reports on fertilizers’ effect on yam production (Enesi et al., 2017). It is presumed that the variation in weather conditions and trial soil fertility status are the possible causes for these conflicting reports (Kang and Wilson, 1981; Lugo et al., 1993; Diby et al., 2011). Varietal differences in fertilizer responses and susceptibility to the low soil fertility might complicate the understanding of application of the fertilizer trial results in yam cultivation. Fertilizer response in yam seems genotype and growth context specific. Hence, genetic improvement efforts need to integrate high yields with high nutrient use efficiency at target farming system (Asiedu and Sartie, 2010). The use of nutrient efficient genotypes should be maximized through breeding and genetic studies to establish environmentally friendly/sustainable agriculture in yam farming system.

We found that the breeding lines performed better than the local variety for low soil fertility tolerance and the added fertilizer response in our experiment. The observed superiority of improved genotypes compared to the local variety ‘Amula’ could be genetic or health status of the planting materials used. Seed degeneration due to virus infection/load is a critical problem with clonally propagated crops like yam. Yield reduction due to virus disease has been reported to be over 40% in yams (Kenyon et al., 2001). The local variety’s viral load might be higher than breeding genotypes used in the experiment as local cultivar’s chronological age is not well known. It has been cultivated and maintained in the field for several years by vegetative propagation, which could have caused the sub-optimal yield performance. The planting materials used in our trial, including the local variety, were visually selected disease-free tubers from disease-free plants. Work done in IITA on a positive selection with seed yam showed a significant decrease in virus incidence and disease severity score while maintaining reasonably good yields (Aihebhoria et al., 2017). However, we did not index the virus status of the planting materials in our trial and acknowledge the sub-optimal yield of local variety could be a confounded effect of the health status of the variety used in the trial.

We noticed an increase in shoot dry weight with added fertilizer, but the impact varied over the years (Figure 2 and Table 2). The difference in fertilizer impact between 2018 and 2019 may be due to different meteorological conditions during field experiments (Figure 2). The temperature was identical in the two years, but the sunshine duration in 2018 was longer than in 2019, mostly from 30 to 90 days after planting when it was shoot growth phase in yam plants (Lebot, 2019). Since the total rainfall in 2019 was higher than in 2018, it could be considered that the utilization efficiency was low due to the outflow of fertilizer by the rain. However, detailed data of meteorological conditions were not recorded and also out of scope this study to quantify nutrient loss by leaching and surface run-off. Attempt was made to apply fertilizer in no rainy days to avoid the chance of excessive nutrient loss from the trial field. Therefore, further investigation is needed to determine type of meteorological factors that influence the fertilizer’s impact on the shoot growth of white Guinea yam.

Our results revealed higher shoot biomass is critical for increased tuber yields in LF and HF conditions (Figure 4). Biomass accumulation and partitioning predicts the tuber yield in yam. Enhanced shoot biomass could support higher tuber yield through the genotype’s efficiency for photosynthate accumulation and partitioning to the storage organ. The positive relationship between fresh tuber yield and shoot dry weight has also been reported (Akoroda, 1984; Sartie et al., 2012) and indicated that vigor biomass might be a morphological marker for selecting genotypes of white Guinea yam for high tuber yield (Sartie et al., 2012). On the other hand, it was suggested that SFSI of shoot growth alone as selection criteria for LF tolerance is not advocated. In genotypes, the SFSI in the shoot growth and the tuber yield was not consistent (Figure 5). Mineral fertilizer effect on yam shoot growth is greatly affected by the growth condition or environment. In contrast, the effect of fertilizer application on tuber yield was greatly affected by the genotypes used (Table 2). Our results, however, were based on limited sample size; hence the further analysis of a large population would provide a better understanding of SFSI of shoot and tuber yield in white Guinea yam. In addition, it has been reported that the predicted value of shoot dry weight using NDVI-based phenotyping method in *D. rotundata* is underestimated when shoot dry weight exceeds 200 g plant^–1^ (Iseki and Matsumoto, 2019). However, the predicted value of shoot dry weight rarely exceeded 200 g plant^–1^ even in HF condition. This is in agreement with the finding from the previous study on *D. rotundata* (Iseki and Matsumoto, 2019). In this sense, the method could also be useful to apply for field evaluation.

In this study, genotypic differences among white Guinea yam (*D. rotundata*) genotypes in response to low soil fertility and added fertilizer input (NPK) were investigated. Future research should include more physiological studies of yam, as there is minimal information available currently. The present study revealed wide variations among the white Guinea yam genotypes for low soil fertility tolerance and added fertilizer response. The genotypes, R034, R041, R050, R052, R060, R100, and R125, were identified as low soil tolerance genotypes in this study. On the other hand, R119, R109, and R131 were susceptible to available low soil nutrient but responsive to mineral fertilizer input. Besides, R025 and R034 have a high potential to produce higher tuber yield than the local variety under LF conditions. The use of superior genotypes identified herewith should be maximized in breeding and genetic studies to develop improved varieties that enhance productivity under variable yam farming systems.

## Data Availability Statement

The original contributions presented in the study are included in the article/Supplementary Material, further inquiries can be directed to the corresponding author/s.

## Author Contributions

RM did the conceptualization, methodology and formal analysis, investigation and fieldwork, and writing-original draft preparation. HI, AA, and RA wrote, reviewed, and edited. HI did the project administration and funding acquisition. All authors read and agreed to the published version of the manuscript.

## Conflict of Interest

The authors declare that the research was conducted in the absence of any commercial or financial relationships that could be construed as a potential conflict of interest.
