# Supplementary figures and images for: Low Soil Nutrient Tolerance and Mineral Fertilizer Response in White Guinea Yam (Dioscorea rotundata) Genotypes
Source: Front Plant Sci. 2021 Feb 19;12:629762. doi: 10.3389/fpls.2021.629762 (PMC7934620; doi:10.3389/fpls.2021.629762)

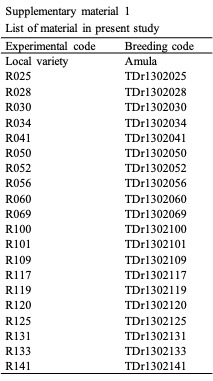

Supplement: Supplementary file 1 [file Image_1.JPEG]

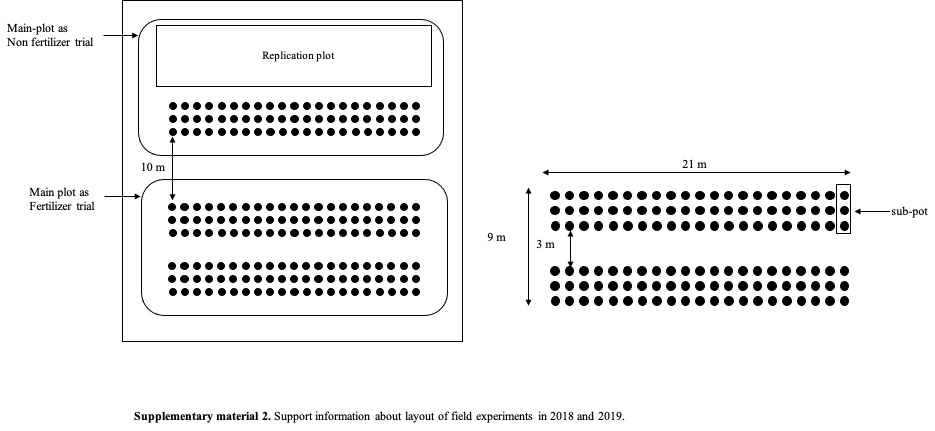

Supplement: Supplementary file 2 [file Image_2.JPEG]
